# The *in vivo* metabolic pathway of Deg-AZM and *in vitro* investigations into the contribution of drug metabolizing enzymes and drug transporters in the drug interactions of Deg-AZM, a clinical-stage new transgelin agonist

**DOI:** 10.3389/fphar.2024.1510903

**Published:** 2025-01-08

**Authors:** Xiaoting Gu, Xiaohe Li, Weixue Tian, Chaoyue Zheng, Yutian Cai, Xiang Xu, Conglu Zhao, Hongting Liu, Yao Sun, Zhilin Luo, Shuwen Zhu, Honggang Zhou, Xiaoyu Ai, Cheng Yang

**Affiliations:** ^1^ State Key Laboratory of Medicinal Chemical Biology, College of Pharmacy and Tianjin Key Laboratory of Molecular Drug Research, Nankai University, Tianjin, China; ^2^ The National Institutes of Pharmaceutical R&D Co., Ltd., Beijing, China; ^3^ Tianjin Key Laboratory of Molecular Drug Research, Tianjin International Joint Academy of Biomedicine, Tianjin, China

**Keywords:** deglycosylated azithromycin, drug-drug interactions, CYP enzymes, Caco-2 cell, P-gp, metabolic pathways

## Abstract

**Introduction:**

Deglycosylated azithromycin (Deg-AZM), a new transgelin agonist with positive therapeutic effects on slow transit constipation, has been approved for clinical trials in 2024. This work investigated the drug metabolism and transport of Deg-AZM to provide research data for further development of Deg-AZM.

**Methods:**

A combination of UPLC-QTOF-MS was used to obtain metabolite spectra of Deg-AZM in plasma, urine, feces and bile. Caco-2 cells was used to investigate the permeability of Deg-AZM and whether it is a potential substrate of the efflux transporter P-glycoprotein. Human liver microsome phenotyping assays with chemical inhibition and recombinant CYPs phenotyping assays were used to investigate the CYP450 enzyme phenotype involved in Deg-AZM metabolism *in vitro*. A HLM inhibition reaction system was established to evaluate the inhibitory effect of Deg-AZM on CYP1A2, CYP2B6, CYP2C8, CYP2C9, CYP2C19, CYP2D6, and CYP3A4. The mRNA expression of human primary hepatocytes incubated with Deg-AZM or not was evaluate the induction of Deg-AZM on CYP1A2, CYP2B6, and CYP3A4.

**Results:**

44 metabolites of Deg-AZM were identified in rat urine, feces, bile, and plasma, the metabolic pathways included demethylation, monohydroxylation, dihydroxylation, dehydroxidation, hydroreduction, hydrolysis, methylation, glucuronidation and the combination of different metabolic pathways. Deg-AZM was a low permeability drug in the intestine and a potential substrate of the efflux transporter P-glycoprotein. CYP3A4 was the major CYP isoform responsible for Deg-AZM metabolism. Deg-AZM showed moderate inhibition with CYP2B6 and CYP2D6. Data in three batches of human primary hepatocytes disclosed induction potential of Deg-AZM on CYP2B6 and CYP3A4.

**Conclusion:**

The *in vivo* metabolic pathway of Deg-AZM and *in vitro* possibility of drug interaction for Deg-AZM with CYP enzymes and drug transporter were fully investigated. It was suggested that dose adjustments may be warranted depending on the potency of the corresponding modulators in clinical.

## 1 Introduction

Chronic constipation manifests as prolonged and repeated obstruction of bowel movements, prolonged defecation time, or difficulty in bowel movement (Costilla and Foxx-Orenstein, 2014; Bharucha and Wald, 2019). It has become a common disease and seriously affects the normal life of patients (Oh et al., 2020). Large statistical analysis showed that the global prevalence of chronic constipation is approximately 15% (Bharucha and Lacy, 2020). A survey in China showed that the incidence rate of chronic constipation is 7.3%–20.39% (Zhi et al., 2021). The current treatment methods for chronic constipation are mainly divided into basic treatment and medication treatment (Rao et al., 2016; Włodarczyk et al., 2021). Basic treatment includes lifestyle adjustment and cognitive therapy (Nunan et al., 2022). The current clinical treatment drugs for chronic constipation include laxatives, volumetric laxatives, osmotic laxatives, irritants, prokinetic laxatives, secretagogues, probiotics/prebiotics, enemas, suppositories, and so on (Tack et al., 2011; Müller-Lissner et al., 2013; El-Salhy et al., 2014; Alsalimy et al., 2018). However, the current treatment methods cannot meet treatment expectations generally, it is still necessary to develop new drugs to treat chronic constipation with high efficiency and low toxicity.

Deglycosylated azithromycin (Deg-AZM) is a new small molecule transgelin agonist independently developed by Nankai University, which has been approved for clinical trials for the treatment of chronic constipation in 2024. Transgelin is the actin-binding protein in the Calponin family, with a relative molecular weight of 22 kDa, mainly distributed in smooth muscle cells (Lees-Miller et al., 1987; Jo et al., 2018; Yin et al., 2018). Deg-AZM can stimulate transgelin on intestinal smooth muscle cells, promote the aggregation of G-actin to F-actin, increase the formation of stress fiber bundles in intestinal smooth muscle cells, promote intestinal peristalsis (Zhong et al., 2020). Deg-AZM has a clear mechanism of action, significant efficacy and high safety. Notably, there are no similar drugs targeting transgelin to treat constipation on the clinical. Deg-AZM is expected to provide a new choice for treating chronic constipation.

More and more patients are using two or more drugs simultaneously, which greatly increases the risk of the potential drug-drug interactions (DDIs) and poses increasing challenges to risk management (Kalyanasundaram et al., 2011; van Leeuwen et al., 2014). Constipation increases with age and often coexists with many diseases, include bowel cancer, cardiovascular diseases, neurological disorders (such as Parkinson’s disease, spinal cord injury, stroke, or multiple sclerosis), and so on (Winge et al., 2003; Ishiyama et al., 2019; De Giorgio et al., 2021). Constipation treatment drugs are often taken together with other drugs and cause DDIs. Exploring DDIs of the new drug candidates is of great significance for the safe use in clinical practice. The major mechanisms of DDIs involve metabolic enzymes and transporters. Therefore, the metabolism and transport-mediated DDIs potential of Deg-AZM need to be thoroughly investigated during development.

The aim of the present study is to investigate the potential mechanisms of metabolic enzymes and transporter associated with Deg-AZM disposition and DDIs. A combination of UPLC-QTOF-MS was used to obtain metabolite spectra of plasma, urine, feces and bile, the structure of the metabolites was inferred, and the proportion of each metabolite of Deg-AZM in the biological sample was calculated according to the peak area. Caco-2 cells was used to investigate the permeability of Deg-AZM and whether it is a potential substrate of the efflux transporter P-glycoprotein (P-gp). Investigation of CYP450 enzyme phenotype involved in Deg-AZM metabolism used human liver microsome (HLM) phenotyping assays with chemical inhibition and Recombinant CYPs phenotyping assays. Establish a HLM inhibition reaction system, measure the concentration of metabolites of seven tool drugs in the system using LC-MS/MS method, IC_50_ was calculated and used to evaluate the inhibitory effect of Deg-AZM on CYP1A2, CYP2B6, CYP2C8, CYP2C9, CYP2C19, CYP2D6, and CYP3A4. The mRNA expression of human primary hepatocytes incubated with Deg-AZM or not was evaluate the induction of Deg-AZM on CYP1A2, CYP2B6, and CYP3A4. This is the first report investigate the potential metabolic enzymes and transporter associated with Deg-AZM.

## 2 Experimental

### 2.1 Chemicals and materials

Deg-AZM was provided by Nankai University (Tianjin, China). Quinidine was obtained from International Laboratories Corp., Ltd. (San Francisco, CA, USA). Rhodamine 123 was purchased from Beijing Solarbio Science & Technology Co., Ltd. (Beijing, China). CMC-Na was provided by Sinopharm Chemical Reagent Co., Ltd. (Shanghai, China). [^3^H] Propranolol and [^14^C] PEG4000 were provided by Perkin Elmer Co. Ltd. (Waltham, MA, USA). Roxithromycin (IS) was provided by the National Institutes for food and drug control (Beijing, China). All reference substances were 98% purity. Methanol, formic acid and acetonitrile with HPLC grade were provided by Thermo Fisher Scientific India Pvt. Ltd. (Waltham, MA, USA). HPLC grade Ammonium acetate was provided by Tianjin Guangfu Technology Development Co., Ltd. (Tianjin, China). Deionized water used throughout the study was provided by Wahaha Corporation (Hangzhou, China).

### 2.2 Metabolism study *in vivo*

#### 2.2.1 Biological sample collection

The plasma samples at 0.167 h, 0.5 h, 3 h and 8 h after administration were obtained from the study of tissue distribution in rats (Gu et al., 2024). Urine, feces and bile samples at 0–24 h after administration were obtained from the excretion study of a single gavage administration of 25 mg/kg Deg-AZM in rats (Gu et al., 2024).

#### 2.2.2 Biological sample preparation

Plasma samples were precipitated with methanol at a ratio of 1:3. Urine samples were diluted with 50% methanol at a ratio of 1:80. The feces was thoroughly ground and sonicated with a 50% methanol at a ratio of 1 g/10 mL to obtain the homogenate, then the homogenate were precipitated with methanol at a ratio of 1:1. Bile samples were precipitated with 50% methanol at a ratio of 1:1. The above obtained samples were vortexed for 1 min and centrifugated at 4°C, 12,000 rpm for 10 min. Finally, the upper layer was analyzed using the UPLC-QTOF-MS system.

#### 2.2.3 UPLC-QTOF-MS conditions

The metabolites of Deg-AZM in the plasma, urine, feces and bile samples were identified by UPLC-QTOF-MS system. Liquid chromatography was performed on an ACQUITY UPLC^TM^ *I*-Class UPLC unit with a Waters ACQUITY UPLC HSS T3 (100 × 2.1 mm, 1.8 µm). A gradient program was employed with the mobile phase combining solvent A (0.02% formic acid in 5% acetonitrile) and solvent B (acetonitrile) as follows: 100% A (0–0.5 min), 100%–85% A (0.5–7 min), 85%–5% A (7–9 min), 5% A (9–12 min), 5%–100% A (12–15 min). The flow rate was 0.40 mL/min. The column temperature was maintained at 40°C.

A Waters Q-TOF Xevo G2-XS Spectrometer equipped with ESI source under positive ion mode was utilized to conduct mass spectrometry detection, for which the MS^E^ mode was set at the mass range of 100–1,000 Da. The cone voltage was 40 V. The desolvation temperature was set at 500°C and source temperature was 100°C. The desolvation gas flow rate was 800 L/h and the cone gas flow rate was 50 L/h. In MS^E^ centroid mode, the MS data were acquired with the low energy function in the trap collision energy (6 eV), and the tandem mass data were acquired with the high energy function in the ramp trap collision energy (35–45 eV).

### 2.3 P-gp transportation and inhibition

#### 2.3.1 Caco-2 cell culture

The human colon adenocarcinoma Caco-2 cell line was provided by Institute of Basic Medicine, Chinese Academy of Medical Sciences (Beijing, China). Caco-2 cells were cultured in a high sugar DMEM medium containing 10% (*v/v*) fetal bovine serum at 37°C and 5% CO_2_ incubator. Caco-2 cells (2.5 ×10^5^/mL) was inoculated into Transwell polycarbonate membrane 12 well plate. The culture medium was changed every 2 days for the first 14 days, and then every day. After 21 consecutive days, fully differentiated cell monolayers were obtained for drug transport assays.

#### 2.3.2 Validation of Caco-2 cell model

Transepithelial electrical resistance (TEER) was used to indicate monolayer integrity. The electrode of the Millcell ERS-2 voltammeter was placed in HBSS solution for 20 min to pre-balance. At the same time, the culture medium was removed from the Transwell culture plate, 0.5 mL of pre-heated HBSS solution was added into the Apical (AP) of transwell, 1.5 mL of pre-heated HBSS solution was added into the Basolateral (BL) of transwell. After that, the transwell was balanced at 37°C for 20 min and then replaced the HBSS solution with the pre-heated HBSS solution, the TEER value was measured finally. Caco-2 cell monolayers with TEER >350 Ω cm^2^ which means that the cells have formed a tight monolayer, were used for the experiment. The TEER value was calculated according to the following formula:

$$\text{TEER}=\left({\text{TEER}}_{T}\;‐\;{\text{TEER}}_{c}\right)\times A$$

where TEER_T_ represented the TEER value (Ω) of the measured Transwell chamber with Caco-2 cells, TEER_C_ was the TEER value (Ω) of the transwell chamber in the control group, and *A* was the membrane area of the transwell cavity (1.12 cm^2^ in this experiment).

In order to evaluate the usability of the Transwell model research, [^3^H] Propranolol (0.5 μCi/mL) and [^14^C] PEG4000 (1 μCi/mL) served as high-permeability and impermeability controls to verify the uptake direction on Caco-2 cell monolayer, respectively. Rhodamine 123 was used as a positive control for efflux mechanism in bidirectional transport assay on Caco-2 cell monolayer.

#### 2.3.3 Drug transport assay

The transmembrane permeability of Deg-AZM (2, 10 or 50 µM) and the potential to inhibit P-gp by Deg-AZM (2, 10 or 50 µM) were evaluated using Caco-2 cell monolayer both apical-to-basolateral (A-B) and basolateral-to-apical (B-A) directions at 37°C for 1 h, the experiments were conducted using quinidine (50 μM) as the inhibitor for P-gp. At the end of incubation, receiver and donor samples were both diluted by assay buffer and analyzed by LC-MS/MS. Bidirectional apparent permeability (*P*_*app*_) of A-B and B-A and the efflux ratios (*P*_*app*_[B-A]/*P*_*app*_[A-B]) were then calculated according to the following formula:

$$P_{app}=\left(V\times dc\right)\;/\;\left(dt\times A\times C_{0}\right)$$

where *V* represented the volume of the solution in the receiving chamber (0.5 cm^3^ at the AP transwell chamber and 1.5 cm^3^ at the BL transwell chamber), *dc* was the concentration of the drug in the receiving chamber, *dt* was the transport time, *A* was the membrane area of the transwell cavity (1.12 cm^2^ at the Transwell membrane used in this experiment), and *C*_*0*_ was the initial concentration.

### 2.4 CYP phenotyping

#### 2.4.1 HLM phenotyping assays

Deg-AZM (1 μM) was treated with pooled HLM (0.5 mg/mL) and NADPH (1 mM) for 90 min with or without seven specific inhibitors (10 µM furafylline for CYP1A2, 10 µM ticlopidine for CYP2B6, 5 µM montelukast for CYP2C8, 10 µM sulfaphenazole for CYP2C9, 10 µM ticlopidine for CYP2C19, 2.5 µM quinidine for CYP2D6, 1 µM ketoconazole for CYP3A). Isoform-selective probe substrates, 10 µM phenacetin for CYP1A2, 20 µM bupropion for CYP2B6, 2 µM amodiaquine for CYP2C8, 5 µM diclofenac for CYP2C9, 20 µM S-mephenytoin for CYP2C19, 5 µM bufuralol for CYP2D6, 2 µM midazolam for CYP3A4, were used as positive controls.

#### 2.4.2 Recombinant CYPs phenotyping assays

Deg-AZM (1 μM) was treated with pooled NADPH (1 mM) and recombinant CYPs (rCYP1A2, rCYP2B6, rCYP2C8, rCYP2C9, rCYP2C19, rCYP2D6, and rCYP3A4, purchased from Research Institute for Liver Diseases Co. Ltd., Shanghai) at 25 pmol/mL for 90 min. The isoform-selective probe substrates served as positive controls (same as the positive controls in HLM phenotyping assays).

### 2.5 CYP inhibition

CYP inhibition potential of Deg-AZM was assessed in NADPH-fortified HLMs (0.5 mg/mL for CYP1A2, CYP2B6, CYP2C19 and CYP2D6 system, 0.035 mg/mL for CYP2C9 system, 0.05 mg/mL for CYP3A4 system, and 0.025 mg/mL for CYP2C8 system) using CYP probe substrates (same as the positive controls in HLM phenotyping assays). Deg-AZM in triplicates (0, 0.2, 0.6, 2.0, 6.0, 20, and 60 μM) was treated with each probe of CYP1A2 (20 min), CYP2B6 (20 min), CYP2C8 (10 min), CYP2C9 (60 min), CYP2C19 (45 min), CYP2D6 (30 min) and CYP3A4 (15 min). Probe substrate metabolites were analyzed by LC-MS/MS and the peak area ratios of analytes to internal standard (Indapamide) were calculated. The activity of the CYP450 enzyme subtype is directly proportional to the concentration of the corresponding probe substrate metabolites. The average of the measured values of each group was calculated, the control tube value was set as 100%, the residual probe substrate metabolites was calculated in the presence of positive inhibitors and Deg-AZM at series concentration. IC_50_ values were calculated by the dose-response model (nonlinear regression) in GraphPad Prism software.

### 2.6 CYP induction

Induction of Deg-AZM on CYP1A2, CYP2B6 and CYP3A4 in three batches of human hepatocytes (MWB, LHuf 17905B, HUB) was assessed by qPCR detection technology. Human hepatocytes were incubated individually with Deg-AZM (5, 25, or 100 μM), a negative control (erythromycin at 10 μM), a positive control (omeprazole at 50 μM for CYP1A2, phenobarbital at 1,000 μM for CYP2B6, or rifampin at 20 μM for CYP3A4) for 24 h, after that, the culture medium was replaced with new one, and hepatocytes was induced for 48 h. After culture and complete induction, total mRNA was extracted by TRNzol RNA Extraction Kit (purchased from Tiangen Biochemical Technology Co., Ltd., Beijing). The RNA was reverse transcribed into cDNA according to the instructions of the Transcriptor First Strand cDNA Synthesis Kit (purchased from Roche, Switzerland). Using reverse transcribed cDNA as a template, amplifying the internal reference genes of β-Actin of the sample and target genes of CYP1A2, CYP2B6, and CYP3A4 respectively to compare the expression differences.

## 3 Results and discussion

### 3.1 Metabolic pathway analysis *in vivo*

**FIGURE 1:**
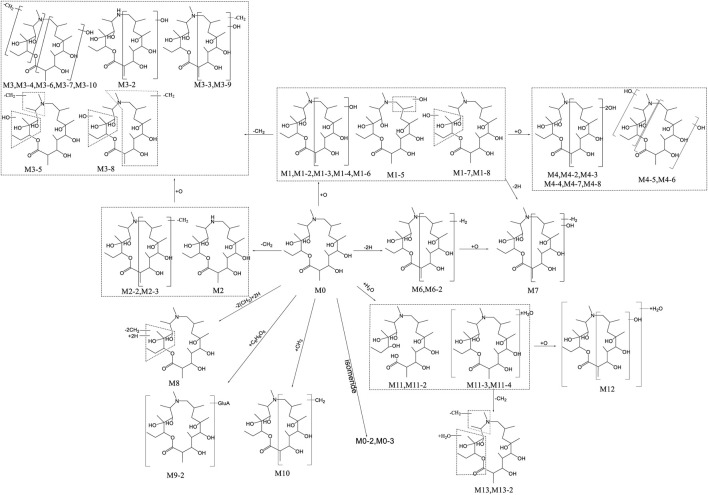
Metabolic pathway analysis in rats after intragastric administration of Deg-AZM.

UPLC-QTOF-MS was applied to analyze blank and post administration urine, feces, bile, and plasma samples of rats. Combined with conventional phase I and II metabolic reaction patterns, a total of 44 metabolites were found in rat urine, feces, bile, and plasma, except for the original form (M0, Deg-AZM). Based on the precise molecular weight and elemental composition of each metabolite, and comparing the fragment ions of the original form and metabolites, the metabolite structure was analyzed. The summary of each metabolite information was shown in Table 1. The main metabolic pathways of Deg-AZM include demethylation, monohydroxylation, dihydroxylation, dehydroxidation, hydroreduction, hydrolysis, methylation, glucuronidation and the combination of different metabolic pathways, as shown in Figure 1.

**TABLE 1:** Metabolites identified in rat plasma and excretion samples after oral administration of Deg-AZM.

Metabolites	Metabolic type	Molecular formula	Retention time (min)	[M + H]^+^ (*m/z*)
M0	Deg-AZM	C_22_H_43_NO_7_	7.07	434.3112
M0-2	Deg-AZM	C_22_H_43_NO_7_	5.34	434.3112
M0-3	Deg-AZM	C_22_H_43_NO_7_	5.45	434.3112
M1	+O	C_22_H_43_NO_8_	5.24	450.3061
M1-2	+O	C_22_H_43_NO_8_	7.38	450.3061
M1-3	+O	C_22_H_43_NO_8_	6.52	450.3061
M1-4	+O	C_22_H_43_NO_8_	8.19	450.3061
M1-5	+O	C_22_H_43_NO_8_	5.59	450.3061
M1-6	+O	C_22_H_43_NO_8_	5.42	450.3061
M1-7	+O	C_22_H_43_NO_8_	4.59	450.3061
M1-8	+O	C_22_H_43_NO_8_	3.14	450.3061
M2	-CH_2_	C_21_H_41_NO_7_	7.10	420.2955
M2-2	-CH_2_	C_21_H_41_NO_7_	5.05	420.2955
M2-3	-CH_2_	C_21_H_41_NO_7_	4.11	420.2955
M3	-CH_2_+O	C_21_H_41_NO_8_	7.22	436.2904
M3-2	-CH_2_+O	C_21_H_41_NO_8_	5.22	436.2904
M3-3	-CH_2_+O	C_21_H_41_NO_8_	5.51	436.2904
M3-4	-CH_2_+O	C_21_H_41_NO_8_	8.15	436.2904
M3-5	-CH_2_+O	C_21_H_41_NO_8_	4.80	436.2904
M3-6	-CH_2_+O	C_21_H_41_NO_8_	6.34	436.2904
M3-7	-CH_2_+O	C_21_H_41_NO_8_	4.15	436.2904
M3-8	-CH_2_+O	C_21_H_41_NO_8_	3.71	436.2904
M3-9	-CH_2_+O	C_21_H_41_NO_8_	5.63	436.2904
M3-10	-CH_2_+O	C_21_H_41_NO_8_	4.44	436.2904
M4	+2O	C_22_H_43_NO_9_	4.74	466.301
M4-2	+2O	C_22_H_43_NO_9_	6.09	466.301
M4-3	+2O	C_22_H_43_NO_9_	4.09	466.301
M4-4	+2O	C_22_H_43_NO_9_	6.41	466.301
M4-5	+2O	C_22_H_43_NO_9_	5.35	466.301
M4-6	+2O	C_22_H_43_NO_9_	3.78	466.301
M4-7	+2O	C_22_H_43_NO_9_	4.39	466.301
M4-8	+2O	C_22_H_43_NO_9_	4.87	466.301
M6	-H_2_	C_22_H_41_NO_7_	7.38	432.2955
M6-2	-H_2_	C_22_H_41_NO_7_	8.19	432.2955
M7	-H_2_+O	C_22_H_41_NO_8_	5.34	448.2904
M8	-2CH_2_+H_2_	C_20_H_41_NO_7_	5.01	408.2955
M9-2	+C_6_H_8_O_6_	C_28_H_51_NO_13_	6.57	610.3433
M10	+CH_2_	C_23_H_45_NO_7_	8.22	448.3269
M11	+H_2_O	C_22_H_45_NO_8_	4.65	452.3218
M11-2	+H_2_O	C_22_H_45_NO_8_	5.69	452.3218
M11-3	+H_2_O	C_22_H_45_NO_8_	6.45	452.3218
M11-4	+H_2_O	C_22_H_45_NO_8_	5.14	452.3218
M12	+H_2_O + O	C_22_H_45_NO_9_	3.64	468.3167
M13	+H_2_O-CH_2_	C_21_H_43_NO_8_	4.36	438.3061
M13-2	+H_2_O-CH_2_	C_21_H_43_NO_8_	5.45	438.3061

Our previous study (Gu et al., 2024) has shown that 8, 8, 9, 13 and 10 metabolites of Deg-AZM were identified from the liver microsomes of mice, rats, dogs, monkeys and humans respectively, the metabolic pathways *in vitro* include hydroxylation, methylation, and combinations of different metabolic pathways. It can be found that all *in vitro* metabolites can be detected in rats. Deg-AZM exhibited more abundant metabolic reactions in rats due to the more complex metabolic enzyme system *in vivo* compared to liver microsomes *in vitro*. The proportion of metabolites *in vivo* and *in vitro* played a crucial role in evaluating the efficacy and toxicity of Deg-AZM, and further research was needed to clarify this.

### 3.2 Exposure profiles of Deg-AZM and metabolites

**FIGURE 2:**
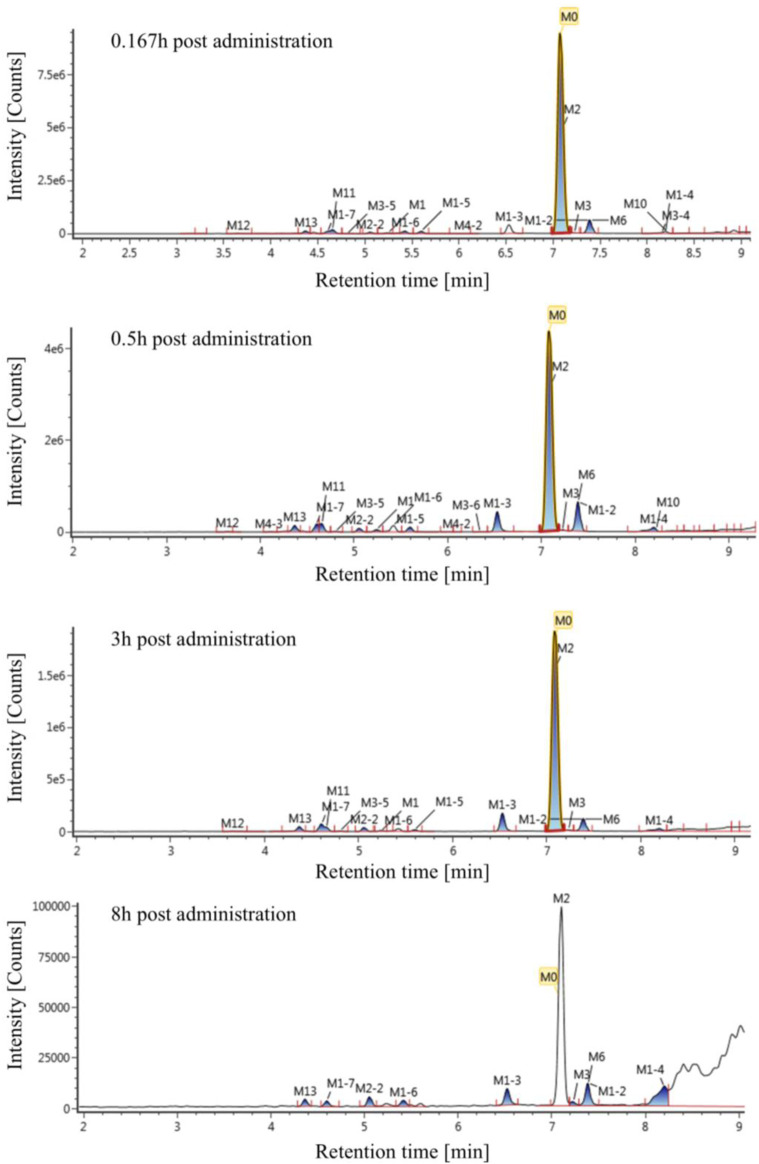
Extraction ion chromatogram of plasma samples at different time points after oral administration of Deg-AZM in rats.

**FIGURE 3:**
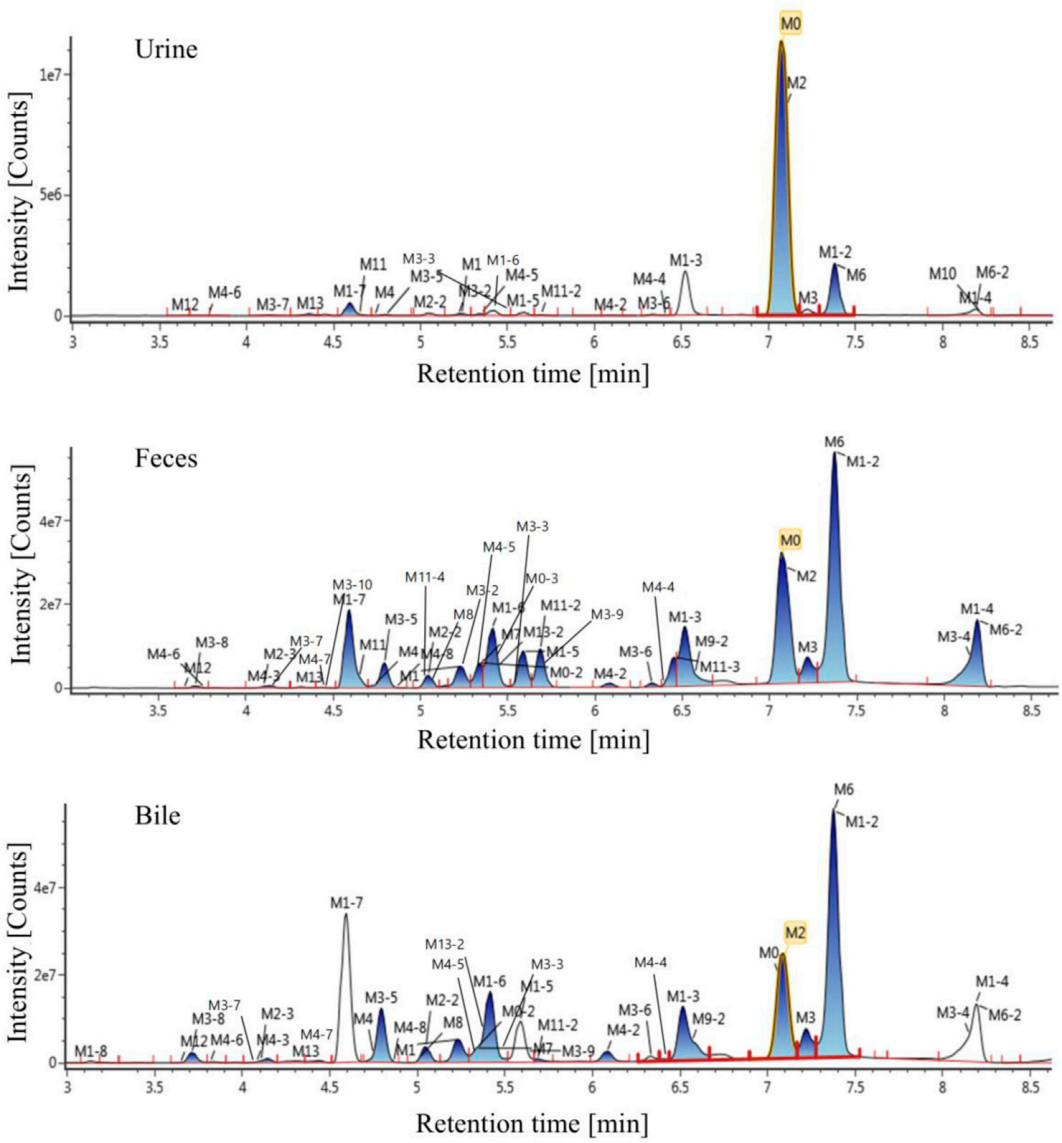
Extraction ion chromatogram of excretion samples after oral administration of Deg-AZM in rats.

**FIGURE 4:**
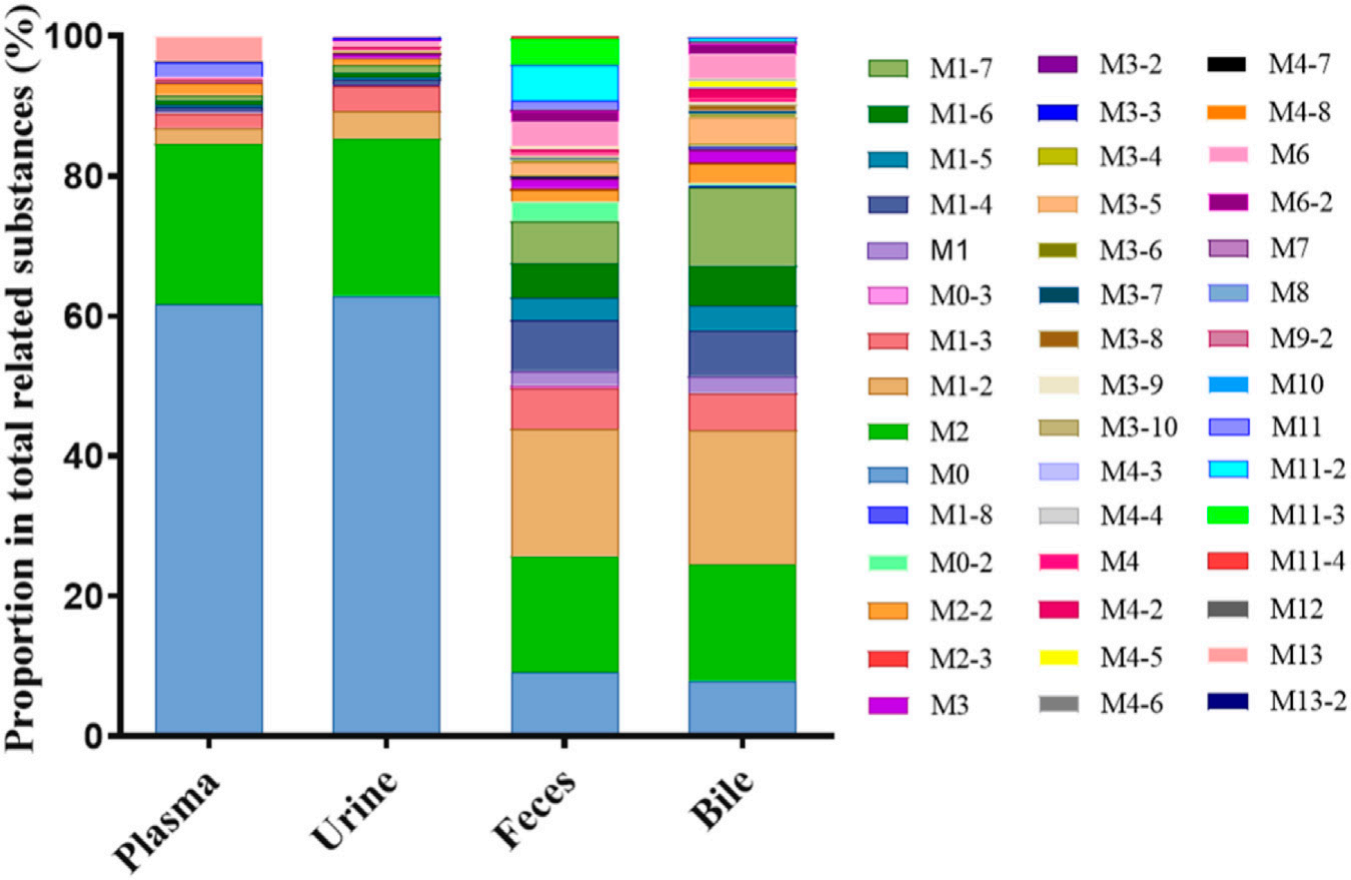
Comparison of exposure profiles of Deg-AZM and its metabolites in plasma, urine, feces, and bile of rats.

The extraction ion chromatogram of plasma samples at 0.167 h, 0.5 h, 3 h and 8 h after administration of Deg-AZM in rats was shown in Figure 2. The extraction ion chromatogram of urine, feces and bile samples collected from 0 to 24 h after oral administration of Deg-AZM in rats was shown in Figure 3. The AUC_0-t_ of Deg-AZM and its metabolites in plasma sample were calculated by trapezoidal rule based on the chromatographic peak area. The relative proportions of Deg-AZM and its metabolites in rat plasma were evaluated by AUC_0-t_, as shown in Figure 4. 21 metabolites were found in rat plasma, with M0 (Deg-AZM) and metabolite M2 (demethylated) being the main exposure forms in rat plasma. M0 and M2 accounted for 61.6% and 23.0% of the total related substances in rat plasma, respectively. The relative proportions of Deg-AZM and its metabolites in urine, feces and bile samples were evaluated by the chromatographic peak area, as shown in Figure 4. The detailed proportion of Deg-AZM and its metabolites could be found in Supplementary Table S1. M0 and 28 metabolites were found in the urine of rats. M0 (62.9%) and metabolite M2 (22.5%) were the main forms of urinary excretion of Deg-AZM in rats after oral administration. M0 and 42 metabolites were found in the feces of rats. Metabolite M1-2 (monohydroxylation, 18.1%) and metabolite M2 (16.6%) were the main forms of fecal excretion of Deg-AZM in rats after oral administration. M0 and 38 metabolites were found in the bile samples of rats. Metabolite M1-2 (monohydroxylation, 19.2%) and metabolite M2 (16.5%) were the main forms of biliary excretion of Deg-AZM in rats after oral administration.

In our previous study (Gu et al., 2024), the excretion experiment in rats (0–96 h) showed that Deg-AZM was mainly excreted and through urine and widely metabolized *in vivo*. The types and proportions of metabolites in the excretion pathway were elucidated in this study. The excretion profile of Deg-AZM in rats (0–24 h) was extracted out showed that the amount of Deg-AZM excreted in its original form from urine and feces was 14.9% and 1.81% of the administered dose, respectively (Gu et al., 2024). Based on the peak area in this study, the total amount of metabolites in urine and feces of rats was 0.589 times and 9.96 times that of the original form of Deg-AZM, respectively. The total amount of Deg-AZM excreted in its original form and various metabolites in urine and feces was 23.7% and 19.8% of the administered dose, respectively, with a material balance coefficient of 43.5%. Deg-AZM was mainly excreted in the form of its original form M0 (cumulative excretion fraction of 14.9%) and metabolite M2 (cumulative excretion fraction of 5.33%) through the kidneys and urine, and in the form of metabolite M1-2 (cumulative excretion fraction of 4.29%) and M2 (cumulative excretion fraction of 3.93%) through the intestines and feces. This study applied high-resolution mass spectrometry to analyze the structure and relative content of the metabolites of Deg-AZM in rats. Due to the differences in mass spectrometry responses between the prototype and metabolites, the peak area normalization method mentioned above was not accurate for the assessment of the excretion ratio of the prototype and metabolites in biological samples. It is necessary to obtain standard samples of the main metabolites for accurate quantification or use isotope tracing techniques to further improve material balance research in the clinical stage.

### 3.3 P-gp substrate and inhibition

#### 3.3.1 Validation of Caco-2 cell model

The TEER value is related to the degree of tight junctions between cells significantly. The larger the value, the tighter the connection between cells. The TEER values of the Caco-2 cell monolayer before administration were all greater than 350 Ω cm^2^ in this study, indicating that the compactness of the Caco-2 cell monolayer meets the experimental requirements.

[^3^H] Propranolol, [^14^C] PEG4000 and Rhodamine 123 were used as tool drugs to test the compactness and efflux function of the Caco-2 cell model. The *P*_*app*_ of [^3^H] Propranolol needed to be less than 27 × 10^−6^ cm/s, and the *P*_*app*_ of [^14^C] PEG4000 needed to be less than 1 × 10^−6^ cm/s. The ER value of Rhodamine 123 without inhibitor of P-gp needed to be greater than 4. As shown in Table 2, The results indicate that the density, integrity, and functionality of the Caco-2 cell monolayer meet the experimental requirements.

**TABLE 2:** *P*_*app*_ values and ER values of bidirectional transport for [^3^H] Propranolol, [^14^C] PEG4000, Rhodamine 123 and Deg-AZM in Caco-2 cell monolayer (n = 3).

Group	*P*_*app*_ (×10^–6^ cm/s)		ER
	AP→BL	BL→AP	
[^3^H] Propranolol	9.64 ± 1.05	—	—
[^14^C] PEG4000	0.199 ± 0.0302	—	—
Rhodamine 123(−)	0.00310 ± 0.00141	0.166 ± 0.0287	53.5
Rhodamine 123 (+)^#^	0.00478 ± 0.000709	0.0417 ± 0.0152	8.73
Deg-AZM (Low-2 μM)	0.326 ± 0.0481	5.27 ± 0.771	16.2
Deg-AZM (Midum-10 μM)	0.302 ± 0.0416	3.70 ± 0.485	12.2
Deg-AZM (High-50 μM)	0.316 ± 0.0201	3.16 ± 0.401	10.0
Deg-AZM (Low-2 μM (+)^#^)	0.823 ± 0.0911**	0.617 ± 0.0488***	0.750
Deg-AZM (Midum-10 μM (+)^#^)	0.471 ± 0.0908*	0.520 ± 0.119***	1.08
Deg-AZM (High-50 μM (+)^#^)	0.589 ± 0.0315***	0.586 ± 0.117***	0.995

^#^ *:* (*+*) *refers to the addition of the inhibitor quinidine.*

—: not applicable.

*p < 0.05.

**p < 0.01.

***p < 0.001, difference before and after quinoline treatment.

#### 3.3.2 Drug transport assay

The bidirectional transport results of the Caco-2 cell monolayer for Deg-AZM at different concentrations were shown in Table 2. At 2, 10 and 50 μM concentration level, the *P*_app_ values of Deg-AZM in the intake direction of Caco-2 model are 0.326 × 10^−6^, 0.302 × 10^−6^ and 0.316 × 10^−6^ cm/s respectively, which imply Deg-AZM was a low permeability drug with poor transmembrane ability. At 2, 10 and 50 μM concentration level, the efflux rates (ER) of Deg-AZM were 16.2, 12.2 and 10.0 respectively, all greater than 2. After the addition of quinidine, the inhibitor of P-gp, the ERs significantly reduced to 0.750, 1.08 and 0.995 respectively. It was inferred that Deg-AZM might be the substrate of intestinal efflux transporter P-gp.

P-gp is an efflux transporter with influencing the pharmacokinetic profiles of many drugs, which expressed on various cells, such as enterocytes, hepatocytes, brain capillary endothelial cells, renal proximal tubular cells (Elmeliegy et al., 2020). In this study, Deg-AZM was inferred to be a substrate of P-gp, which suggested that we should pay attention on the systemic exposure of Deg-AZM when combined with the inhibitors or inducers of P-gp in the clinical trials. A significant change in system exposure of the drug may lead to toxicity or insufficient efficacy. This study did not clarify whether Deg-AZM is an inducer or inhibitor of P-gp, and we need to continue researching to provide more guidance for the management of DDIs study in clinical trials.

### 3.4 CYP phenotyping

#### 3.4.1 HLM phenotyping assays

The inhibition rates of the generation of corresponding metabolites in seven positive control reaction systems were investigated. Compared with the control groups without inhibitors, specific inhibitors significantly inhibited the generation of corresponding products of each probe substrate, with inhibition rates ranging from 83.1% to 94.9%, confirming the effectiveness of the experimental system.

**FIGURE 5:**
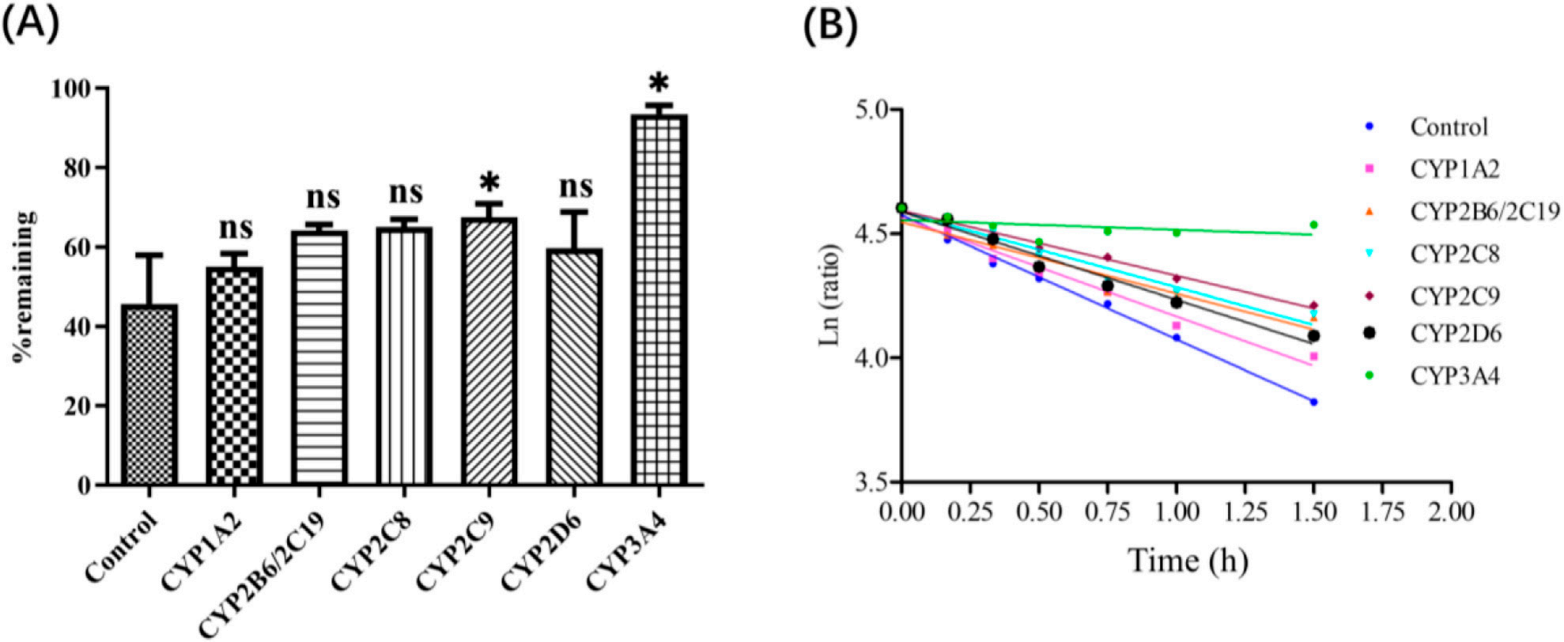
**(A)**: Deg-AZM remaining after incubation in HLM with chemical inhibitors. **(B)**: Half log fitting plot of Deg-AZM remaining at different time after incubation in HLM with chemical inhibitors. Data were presented as the mean ± SD, n = 3. ns: no significance; ^*^ *p* < 0.05, compared to the control group.

Deg-AZM phenotyping of CYPs was evaluated by HLMs with specific inhibitors. After 90 min incubation, 45.7% Deg-AZM remained in the control group without inhibitors, indicating that Deg-AZM undergoes metabolic with CYPs. The addition of the CYP3A inhibitor ketoconazole resulted in 93.4% of Deg-AZM remaining, the addition of the CYP2C9 inhibitor sulfaphenazole resulted in 67.5% of Deg-AZM remaining, while the addition of all the other CYP inhibitors produced nearly no effect (Figure 5). It indicated that CYP3A4 mainly involved in the metabolic of Deg-AZM, CYP2C9 may be involved in the metabolic of some Deg-AZM.

The above results showed that 45.7% Deg-AZM remained in the control group without inhibitors, but the addition of the CYP3A inhibitor ketoconazole resulted in 93.4% of Deg-AZM remaining, this contribution rate was already significant enough, so we did not verify the consumption of the inhibitors in the incubation system further. However, the disappearance of the inhibitors may affect their ability to block CYP-specific metabolism, which will lead to underestimation the contribution of the corresponding metabolic enzyme. In addition, the sufficient specificity of inhibitors also affects the accuracy of the results. Therefore, “Recombinant CYPs phenotyping assay” needed to be employed to verified the results of CYPs phenotyping of Deg-AZM.

#### 3.4.2 Recombinant CYPs phenotyping assays

**FIGURE 6:**
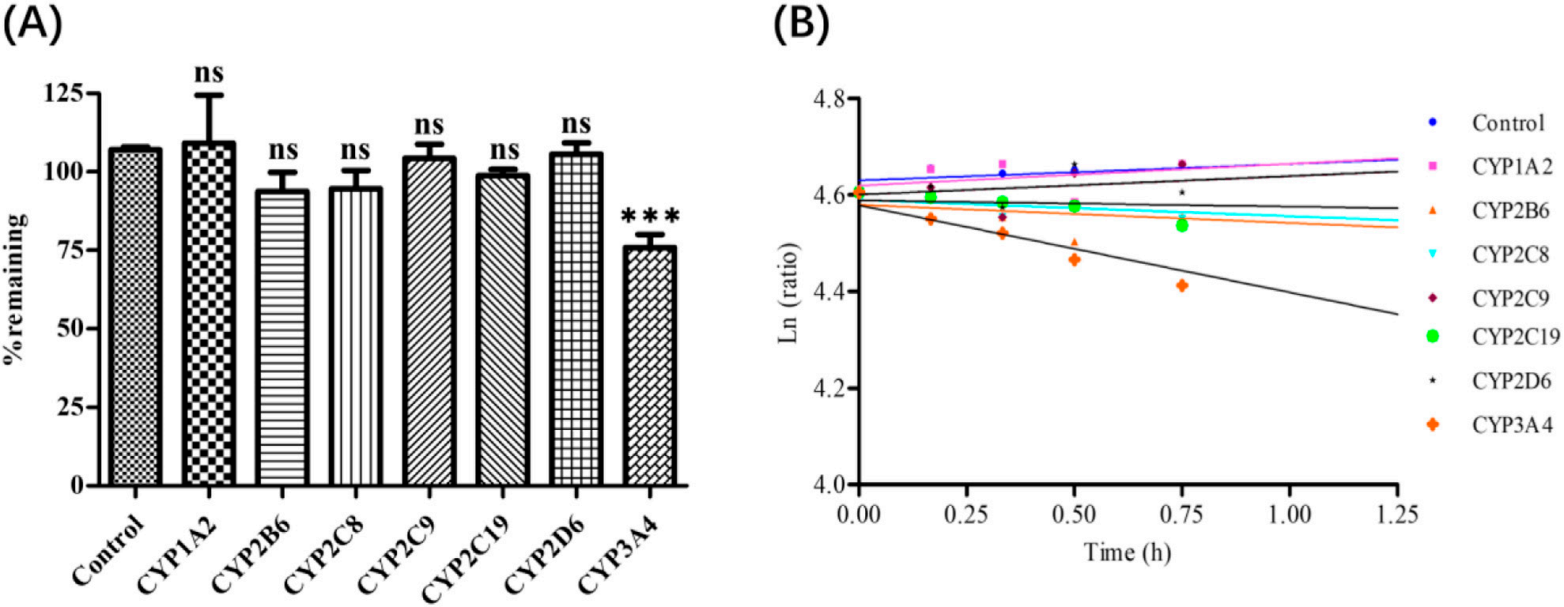
**(A)**: Deg-AZM remaining after incubation in recombinant enzymes. **(B)**: Half log fitting plot of Deg-AZM remaining at different time after incubation in recombinant enzymes. Data were presented as the mean ± SD, n = 3. ns: no significance; ^***^ *p* < 0.001, compared to the control group.

Deg-AZM phenotyping of CYPs was evaluated by rCYPs ulteriorly. The remaining proportion of Deg-AZM in each recombinant CYP monomer enzyme after incubation with Deg-AZM was shown in Figure 6A, and the average remaining proportion time semi logarithmic fitting curve was shown in Figure 6B. The results showed that the residual proportion of Deg-AZM in the control group with inactivated monomeric enzyme after 1.5 h of incubation was 107%, indicating that Deg-AZM was stable in the reaction system without significant non enzymatic metabolic elimination. Among the seven active experimental groups, after incubation for 1.5 h, the average residual proportion of Deg-AZM in the CYP3A4 monomeric enzyme system was 75.9%, the elimination proportion was greater than 15%, and the *k*e value was higher than that of the blank control group and other monomeric enzyme groups. There was no significant difference for the elimination proportion and *k*e value of Deg-AZM in other monomeric enzyme systems compared with the blank control group. In summary, only rCYP3A4 led to a remarkable disappearance of Deg-AZM.

Overall, the results of “*HLM phenotyping assays*” and “*Recombinant CYPs phenotyping assays”* showed that CYP3A4 is the main enzyme subtype mediating Deg-AZM liver metabolism, CYP2C8, CYP2D6, CYP1A2, CYP2B6, CYP2C9 and CYP2C19 did not participate in the metabolism of Deg-AZM.

### 3.5 CYP inhibition

The IC_50_ values of Deg-AZM against CYP1A2, CYP2B6, CYP2C8, CYP2C9, CYP2C19, CYP2D6, and CYP3A4 in HLMs was shown in Table 3. Deg-AZM has no inhibitory effect on CYP1A2, CYP2C8, CYP2C9, CYP2C19 and CYP3A4 with IC_50_ values greater than the maximum concentration set in the experiment of 60 μM, but has a moderate inhibitory effect on CYP2B6 and CYP2D6 with IC_50_ of 8.97 μM and 7.89 μM respectively.

**TABLE 3:** Evaluation of Deg-AZM as an inhibitor of CYPs in HLMs.

CYPs	CYP1A2	CYP2B6	CYP2C8	CYP2C9	CYP2C19	CYP2D6	CYP3A4
IC_50_ (μM)	>60	8.97	>60	>60	>60	7.89	>60

The single probe substrate combined with LC-MS/MS method is the “gold standard” for *in vitro* evaluation of CYP inhibition. However, this method still has some limitations. In this study, Deg-AZM was used for pre-incubation up to 60 min with liver microsomes in some group to inhibit the activity of CYP enzymes. Our previous study (Gu et al., 2024) has shown that Deg-AZM was not stable in human liver microsomes (T_1/2_ = 46.5 min), which indicated that the content of Deg-AZM decreased in the system with incubation time increasing, which may lead to an underestimation of the inhibitory strength of Deg-AZM on some CYP enzymes. Further exploration is needed in subsequent clinical studies.

### 3.6 CYP induction

Induction results of positive control, negative control and Deg-AZM on CYP1A2, CYP2B6 and CYP3A4 in three batches of human hepatocytes were listed in Table 4. Positive inducers omeprazole, phenobarbital and rifampicin significantly induced mRNA expression of CYP1A2, CYP2B6 and CYP3A4 in human hepatocytes, respectively. The negative control group (Erythromycin) showed no significant effect on the mRNA expression levels of CYP1A2, CYP2B6, and CYP3A4 in human hepatocytes. Deg-AZM at 5, 25, and 100 µM showed no induction effect on CYP1A2. At 5, 25, and 100 µM concentration levels, the induction of Deg-AZM on CYP2B6 in MWB batch of human hepatocyte were 1.60, 1.83, and 4.33 times higher than those of the control group, and 4.39%, 6.09% and 24.3% (over 20%) of the positive control, respectively. The induction of Deg-AZM on CYP2B6 in LHuf17905B batch of human hepatocyte were 1.61, 1.46, and 2.84 times higher than that of the control group, and 6.61%, 5.00% and 19.8% of the positive control, respectively. Overall, Deg-AZM has an induction effect on CYP2B6 enzyme. At 5, 25, and 100 µM concentration levels, the induction of Deg-AZM on CYP3A4 in three batches of human hepatocytes showed a significant concentration dependent trend. The induction of Deg-AZM on CYP3A4 in MWB batch of human hepatocyte were 1.96, 4.05, and 15.2 times higher than those of the control group, and 1.99%, 6.32% and 29.3% (over 20%) of the positive control, respectively. The induction of Deg-AZM on CYP3A4 in LHuf17905B batch of human hepatocyte were 2.30, 3.84, and 13.7 times higher than those of the control group, and 5.55%, 12.1% and 54.2% (over 20%) of the positive control, respectively. The induction of Deg-AZM on CYP3A4 in HUB batch of human hepatocyte were 1.80, 2.13 and 6.01 times higher than those of the control group, and 2.15%, 3.06% and 13.5% of the positive control, respectively. Based on the above results, Deg-AZM has an inducing effect on the CYP3A4 enzyme in primary human hepatocytes.

**TABLE 4:** Induction of Deg-AZM on CYP1A2, CYP2B6 and CYP3A4 in three batches of human hepatocytes (n = 3).

Compound	Concentration	CYP1A2						CYP2B6						CYP3A4						
		MWB	% of PC	LHuf 17905B	% of PC	HUB	% of PC	MWB	% of PC	LHuf 17905B	% of PC	HUB	% of PC	MWB		% of PC	LHuf 17905B	% of PC	HUB	% of PC
Deg-AZM	5 μM	1.02	0.0293	0.721	−0.507	0.850	−0.318	1.60	4.39	1.61	6.61	0.942	−0.466	1.96		1.99	2.30	5.55	1.80	2.15
	25 μM	0.948	−0.0814	0.687	−0.569	0.835	−0.349	1.83	6.09	1.46	5.00	1.00	0.0278	4.05		6.32	3.84	12.1	2.13	3.06
	100 μM	0.797	−0.319	0.885	−0.209	1.03	0.0545	4.33	24.3	2.84	19.8	1.37	2.97	15.2		29.3	13.7	54.2	6.01	13.5
Omeprazole	50 μM	64.6	—	56.0	—	48.2	—	—	—	—	—	—	—	—		—	—	—	—	—
Phenobarbital	1 mM	—	—	—	—	—	—	14.7	—	10.3	—	13.5	—	—		—	—	—	—	—
Rifampicin	20 μM	—	—	—	—	—	—	—	—	—	—	—	—	49.3		—	24.4	—	38.1	—
Erythromycin	10 μM	1.15	0.230	0.602	−0.723	0.969	−0.0650	1.31	2.25	1.25	2.74	0.826	−1.39	1.46		0.961	1.89	3.78	1.24	0.646

PC: positive control.

—: not applicable.

According to FDA guidelines (Huang et al., 2007), CYP2D6 has not been shown to be inducible, while CYP2C and CYP2B have synergistic inducing effects with CYP3A, which appears to be sensitive to all known synergistic inducers. Therefore, in order to evaluate whether the investigational drug has an inducing effect on CYP1A2, CYP2C8, CYP2C9, CYP2C19 or CYP3A, the initial *in vitro* induction evaluation may only include CYP1A2 and CYP3A. If the results of *in vitro* studies indicate that the investigational drug does not induce CYP3A metabolism, then there is no need to conduct *in vivo* interaction studies between the investigational drug and co-administered drugs eliminated by CYP2C/CYP2B and CYP3A at these enzyme induction levels. It is important to investigate the DDIs mediated by CYP2B6 in both preclinical and clinical stages. Other CYP enzymes including CYP2A6 and CYP2E1 are considered to be less involved in clinically DDIs. Therefore, this study conducted the *in vitro* induction study of Deg-AZM on CYP1A2, CYP2B6 and CYP3A of human hepatocytes. This study indicated that Deg-AZM has an inducing effect on the CYP3A4 enzyme, suggesting that we should pay attention to plasma exposure of the combination drugs to avoid drug failure caused by low blood concentration or toxicity caused by the toxic metabolites in clinical application.

## 4 Conclusion

This study elaborated the metabolic pathway of Deg-AZM *in vivo* and revealed the potential role of Deg-AZM as a perpetrator of DDIs by regulating metabolic enzymes or transporter *in vitro* firstly. Deg-AZM and a total of 44 metabolites were found in rat urine, feces, bile, and plasma. The main metabolic pathways of Deg-AZM include demethylation, monohydroxylation, dihydroxylation, dehydroxidation, hydroreduction, hydrolysis, methylation, glucuronidation and the combination of different metabolic pathways. Deg-AZM was a potential substrate of the efflux transporter P-gp. CYP3A4 is the major CYP isoform responsible for Deg-AZM metabolism, the exposure of Deg-AZM could be impacted by CYP3A4 modulators. Deg-AZM showed moderate inhibition for CYP2B6 and CYP2D6. Data in human primary hepatocytes disclosed induction potential of Deg-AZM on CYP2B6 and CYP3A4. These *in vitro* results with CYPs and transporter indicated that CYP3A and P-gp inhibitors and inducers may impact systemic exposure of Deg-AZM and caused DDIs.

## Data Availability

The raw data supporting the conclusions of this article will be made available by the authors, without undue reservation.

## Abbreviations

AP, Apical; BL, Basolateral; Caco-2, the human colon adenocarcinoma cell line; CYP, cytochrome P450; DDIs, drug-drug interactions; Deg-AZM, Deglycosylated azithromycin; ER, efflux ratio; HBSS, Hank’s balanced salt solution; HLM, human liver microsome; HPLC, High performance liquid chromatography; IC_50_, half maximal inhibitory concentration; LC-MS/MS, liquid chromatography coupled with tandem; NADPH, nicotinamide adenine dinucleotide phosphate; *P*_*app*_, apparent permeability coefficient; P-gp, P-glycoprotein; SD, standard deviation; TEER, Transepithelial electrical resistance.
