# Supplementary material for: The in vivo metabolic pathway of Deg-AZM and in vitro investigations into the contribution of drug metabolizing enzymes and drug transporters in the drug interactions of Deg-AZM, a clinical-stage new transgelin agonist
Source: Front Pharmacol. 2025 Jan 8;15:1510903. doi: 10.3389/fphar.2024.1510903 (PMC11750672; doi:10.3389/fphar.2024.1510903)
Supplement: Supplementary file 1 [file Table1.docx]

Table S1. Comparison of exposure profiles of Deg-AZM and its metabolites in plasma, urine, feces, and bile of rats.

| Metabolites | molecular formula | Proportion in total related substances (%) | | | |
| --- | --- | --- | --- | --- | --- |
|  |  | Plasma | Urine | Feces | Bile |
| M0 | C_22_H_43_NO_7_ | 61.6 | 62.9 | 9.12 | 7.95 |
| M0-2 | C_22_H_43_NO_7_ | / | / | 2.74 | 0.446 |
| M0-3 | C_22_H_43_NO_7_ | / | / | 0.460 | / |
| M1 | C_22_H_43_NO_8_ | 0.219 | 0.201 | 2.05 | 2.35 |
| M1-2 | C_22_H_43_NO_8_ | 2.26 | 3.84 | 18.1 | 19.2 |
| M1-3 | C_22_H_43_NO_8_ | 2.12 | 3.52 | 5.85 | 5.38 |
| M1-4 | C_22_H_43_NO_8_ | 0.558 | 0.842 | 7.26 | 6.60 |
| M1-5 | C_22_H_43_NO_8_ | 0.392 | 0.376 | 3.18 | 3.50 |
| M1-6 | C_22_H_43_NO_8_ | 0.563 | 0.546 | 4.89 | 5.73 |
| M1-7 | C_22_H_43_NO_8_ | 0.912 | 1.17 | 6.02 | 11.1 |
| M1-8 | C_22_H_43_NO_8_ | / | / | / | 0.255 |
| M2 | C_21_H_41_NO_7_ | 23.0 | 22.5 | 16.6 | 16.5 |
| M2-2 | C_21_H_41_NO_7_ | 1.57 | 0.923 | 1.73 | 2.70 |
| M2-3 | C_21_H_41_NO_7_ | / | / | 0.240 | 0.215 |
| M3 | C_21_H_41_NO_8_ | 0.293 | 0.589 | 1.29 | 1.68 |
| M3-2 | C_21_H_41_NO_8_ | / | 0.0890 | 0.262 | 0.309 |
| M3-3 | C_21_H_41_NO_8_ | 0.0114 | 0.0231 | 0.142 | 0.327 |
| M3-4 | C_21_H_41_NO_8_ | 0.00353 | / | 0.168 | 0.287 |
| M3-5 | C_21_H_41_NO_8_ | 0.173 | 0.182 | 1.81 | 3.87 |
| M3-6 | C_21_H_41_NO_8_ | 0.0198 | 0.194 | 0.367 | 0.510 |
| M3-7 | C_21_H_41_NO_8_ | / | 0.0625 | 0.247 | 0.453 |
| M3-8 | C_21_H_41_NO_8_ | / | / | 0.203 | 0.876 |
| M3-9 | C_21_H_41_NO_8_ | / | / | 0.126 | 0.334 |
| M3-10 | C_21_H_41_NO_8_ | / | / | 0.0991 | / |
| M4 | C_22_H_43_NO_9_ | / | 0.0320 | 0.342 | 0.523 |
| M4-2 | C_22_H_43_NO_9_ | 0.0203 | 0.0493 | 0.535 | 1.38 |
| M4-3 | C_22_H_43_NO_9_ | 0.0116 | / | 0.0616 | 0.127 |
| M4-4 | C_22_H_43_NO_9_ | / | 0.0276 | 0.0327 | 0.117 |
| M4-5 | C_22_H_43_NO_9_ | / | 0.0234 | 0.196 | 0.904 |
| M4-6 | C_22_H_43_NO_9_ | / | 0.0130 | 0.0435 | 0.0836 |
| M4-7 | C_22_H_43_NO_9_ | / | / | 0.0374 | 0.101 |
| M4-8 | C_22_H_43_NO_9_ | / | / | 0.0637 | 0.0953 |
| M6 | C_22_H_41_NO_7_ | 0.379 | 0.634 | 3.52 | 3.49 |
| M6-2 | C_22_H_41_NO_7_ | / | 0.207 | 1.56 | 1.44 |
| M7 | C_22_H_41_NO_8_ | / | / | 0.0787 | 0.324 |
| M8 | C_20_H_41_NO_7_ | / | / | 0.0400 | 0.0660 |
| M9-2 | C_28_H_51_NO_13_ | / | / | 0.00949 | 0.0292 |
| M10 | C_23_H_45_NO_7_ | 0.0155 | 0.0343 | / | / |
| M11 | C_22_H_45_NO_8_ | 2.04 | 0.224 | 1.24 | / |
| M11-2 | C_22_H_45_NO_8_ | / | 0.149 | 5.09 | 0.481 |
| M11-3 | C_22_H_45_NO_8_ | / | / | 3.78 | / |
| M11-4 | C_22_H_45_NO_8_ | / | / | 0.305 | / |
| M12 | C_22_H_45_NO_9_ | 0.296 | 0.0529 | 0.0202 | 0.0846 |
| M13 | C_21_H_43_NO_8_ | 3.51 | 0.558 | 0.0619 | 0.0987 |
| M13-2 | C_21_H_43_NO_8_ | / | 0.0580 | 0.0395 | 0.112 |

*/: not detected*
